# Resources availability and COVID-19 mortality among US counties

**DOI:** 10.3389/fpubh.2023.1098571

**Published:** 2023-03-01

**Authors:** Josué Patien Epané, Ferhat Zengul, Zo Ramamonjiarivelo, Luceta McRoy, Robert Weech-Maldonado

**Affiliations:** ^1^Department of Healthcare Administration, School of Public Health, Loma Linda University, Loma Linda, CA, United States; ^2^Department of Health Services Administration, School of Health Professions, University of Alabama at Birmingham, Birmingham, AL, United States; ^3^School of Health Administration, College of Health Professions, Texas State University, San Marcos, TX, United States; ^4^College of Business, Lander University, Greenwood, SC, United States

**Keywords:** COVID-19, mortality, resource availability, US hospitals, US counties

## Abstract

The COVID-19 was declared a pandemic by WHO on 03/2020 has claimed millions of lives worldwide. The US leads all countries in COVID-19-related deaths. Individual level (preexisting conditions and demographics) and county-level (availability of resources) factors have been attributed to increased risk of COVID-19-related deaths. This study builds on previous studies to assess the relationship between county-level resources and COVID-19 mortality among 2,438 US counties. We merged 2019 data from AHA, AHRF, and USA FACTS. The dependent variable was the total number of COVID-19-related deaths. Independent variables included county-level resources: (1) hospital staffing levels (FTE RNs, hospitalists, and intensivists) per 10,000 population; (2) hospital capacity (occupancy rate, proportion of teaching hospitals, and number of airborne infection control rooms per 10,000 population); and (3) macroeconomic resources [per capita income and location (urban/rural)]. We controlled for population 65+, racial/ethnic minority, and COVID-19 deaths per 1,000 population. A negative binomial regression was used. Hospital staffing per 10,000 population {FTE RN [IRR = 0.997; CI (0.995–0.999)], FTE hospitalists [IRR = 0.936; CI (0.897–0.978)], and FTE intensivists [IRR = 0.606; CI (0.516–0.712)]} was associated with lower COVID-19-related deaths. Hospital occupancy rate, proportion of teaching hospitals, and total number of airborne infection control rooms per 10,000 population were positively associated with COVID-19-related deaths. Per capita income and being in an urban county were positively associated with COVID-19-related deaths. Finally, the proportion of 65+, racial/ethnic minorities, and the number of cases were positively associated with COVID-19-related deaths. Our findings suggest that focusing on maintaining adequate hospital staffing could improve COVID-19 mortality.

## 1. Introduction

As of February 10, 2022, there have been over 400 million confirmed COVID-19 cases and over 5 million COVID-19-related deaths worldwide (1), with the United States having the highest number of COVID-19 cases (over 76 million) and deaths (over 900 thousand) of any country (2). COVID-19 treatment is highly resource intensive and has overwhelmed healthcare systems, especially in pandemic-ridden geographic areas (3). Healthcare resources are defined as “the labor and capital devoted to healthcare, i.e., healthcare workers, healthcare facilities, financial resources, and knowledge, as well as the equipment and materials used to deliver healthcare services” (4). Based on that definition, the availability of healthcare resources increases access to care, and consequently affects health outcomes (4). As such, the availability of healthcare resources to treat COVID-19 patients plays a major role in patients' health outcomes. The purpose of this study is to assess the association between healthcare resource availability and COVID-19 mortality in US counties.

## 2. Literature review

Several studies have examined the relationship between healthcare resources and COVID-19-related deaths. Janke et al. assessed hospital resource availability and COVID-19 mortality among hospital referral regions in the US and found a negative relationship between resource availability, in terms of intensive care unit beds, nurses, and general medicine/surgical beds, and COVID-19-related deaths (3). Findings regarding the relationship between COVID-19-related deaths and county-level health-related resources are not conclusive. One study found that the availability of resources with regards to the number of physicians per capita was associated with a decreased death rate (5). Similarly, another study found that the availability of resources in terms of the number of acute care beds was associated with a reduced death rate (6). However, another study found that an increased number of acute care beds per capita was associated with increased mortality rate (5).

Other studies have examined the association between county-level COVID-19 mortality rate and health status of county residents, county-level demographic and macroeconomic environment, county-level political association, and county-level health-associated resources. There are some positive associations between county COVID-19-related death rates and the percentage of the county population who drink alcohol, have asthma, Alzheimer's, depression, diabetes, and stroke (6). Some studies have indicated that the COVID-19-related death rate has a positive relationship with county population density (6); the percentage of the minority population in the county, namely Native American, Black, Asian, and Hispanic (5–7); as well as percentage of unemployed individuals, percentage of individuals in poverty in the county, and county-level democratic tendency (6). Higher county median household income was associated with a lower COVID-19-related death rate.

Other factors have been found to increase the risk of COVID-19 mortality as evidenced by extant empirical studies. Factors such as demographic profile and pre-existing conditions are risk factors for COVID-19-related deaths. Several studies have indicated that older patients (7–11), male patients (7, 9, 11), Black patients (8, 11), Asian or Pacific Islander patients (11), American Indian or Alaska native patients (8), and patients of Hispanic ethnicity (8, 12) are more likely to die of COVID-19, compared with their younger, female, and White non-Hispanic counterparts. Patients with pre-existing conditions such as cancer (10), lung diseases (7, 9, 11), diabetes (7, 9, 11), high cholesterol level (7), cardiovascular diseases (9), and neurologic disorders (9) have higher mortality rate than COVID-19 patients without pre-existing conditions.

Saving lives by treating diseases and healing patients require adequate healthcare resources. However, given the unprecedented increase in demand for resources during an outbreak, resources may become suddenly scarce. Dealing with a pandemic with depleted resources may also cause hospitals to cancel elective procedures, which may contribute to a significant loss in patient revenue (13). The aim of this study is to build on COVID-19 empirical literature by assessing the relationship between the level of healthcare resources and COVID-19 mortality rate among US counties (1).

## 3. Conceptual framework

The characteristics of healthcare delivery systems, such as resources and organizational structure, are major determinants of health services utilization/access to care (4). Access to healthcare services may increase the probability of having a positive health outcome (recovery from illness). Healthcare resources include the labor force, infrastructure/facilities/departments/, and services offered. For instance, a hospital needs to hire clinicians, such as hospitalists and nurses, build specific healthcare facilities, and purchase equipment for specific disease and services. The availability of such resources has been found to mitigate the number of COVID-19-related deaths and COVID-19 outbreaks in nursing homes (14). The other type of resources consists of the macroeconomic resources that the organization needs. For instance, areas with higher per capita income possess more financial resources to support healthcare organizations than areas with a low-income population. Income inequalities in US counties have been found to have a positive relationship with COVID-19 cases and related deaths (15).

Since the availability of resources enables hospitals to deliver healthcare services, and access to healthcare services increases the probability of positive health outcomes, it is hypothesized that:

Hypothesis: There is a negative relationship between resource availability and COVID-19-related deaths.

## 4. Materials and methods

### 4.1. Data

Data used for this study were derived from the 2019 American Hospital Association (AHA) Annual Survey, the Area Health Resources Files (AHRF), and USA FACTS (COVID-19-related cases and deaths as of December 14, 2022). The AHA survey provides data on utilization, hospitals' staffing, and organizational structure (16). The AHRF provides county-level data on population characteristics and resource availability (17). The USA FACTS contains data pertaining to US government finances, security, and safety, macroeconomy, as well as population and demographics, education, and health. We retrieved data on COVID-19-related deaths from USA FACTS (18). Our study was deemed exempt for human subjects review by the Institutional Review Board of the University of Nevada, Las Vegas.

Our cross-sectional study sample consists of all US counties that reported any COVID-19-related death (including 0) between February 6th, 2020, and February 13th, 2022. Our final sample consisted of 2,438 counties. As such, establishing causality, or addressing reverse causality was beyond the scope of our study.

### 4.2. Variables

The dependent variable used for this analysis was the total number of COVID-19-related deaths in the county from February 6th, 2020, to February 13th, 2022. The independent variables included county-level hospital resources and macroeconomic resources associated with COVID-19-related deaths. County-level hospital resources extracted from the AHA Annual Survey data included the number of full-time equivalent (FTE) registered nurses (RN) per 10,000 population, hospitalists per 10,000 population, intensivists per 10,000 population, airborne infection control rooms per 10,000 population, the average hospital occupancy rate, and the proportion of hospitals that are teaching hospitals. These measures are believed to be more likely to have an impact on COVID-19-related deaths (3). The county-level macroeconomic resources extracted from the AHRF data included the county-level per capita income and whether the county is urban or rural. We also controlled for the percentage of the population 65 years and older, the percent of racial/ethnic minorities in the county, and the number of COVID-19 cases per 1,000 population in the county, since these variables have been found to be associated to COVID-19-related deaths (19).

### 4.3. Analysis

We used univariate analyses to obtain the descriptive for the dependent, independent, and control variables. Given the overdispersed count data of the dependent variable, county level COVID-19-related deaths, a negative binomial regression was used to model the relationship between the dependent variable and hospital resources, macroeconomic resources, and control variables. Overdispersion is when the variance is greater than the mean, and in this case the conditional variance exceeded over 3,000 times the conditional mean of the dependent variable. According to Fávero et al., given that the variance exceeds the mean, a negative binomial regression is more appropriate than a Poisson regression (20). SAS Version 9.4 was used for data cleaning, and STATA Version 13 was used for data analysis. Statistical significance was established at a *p*-value ≤ 0.05.

## 5. Results

**Figure 1:**
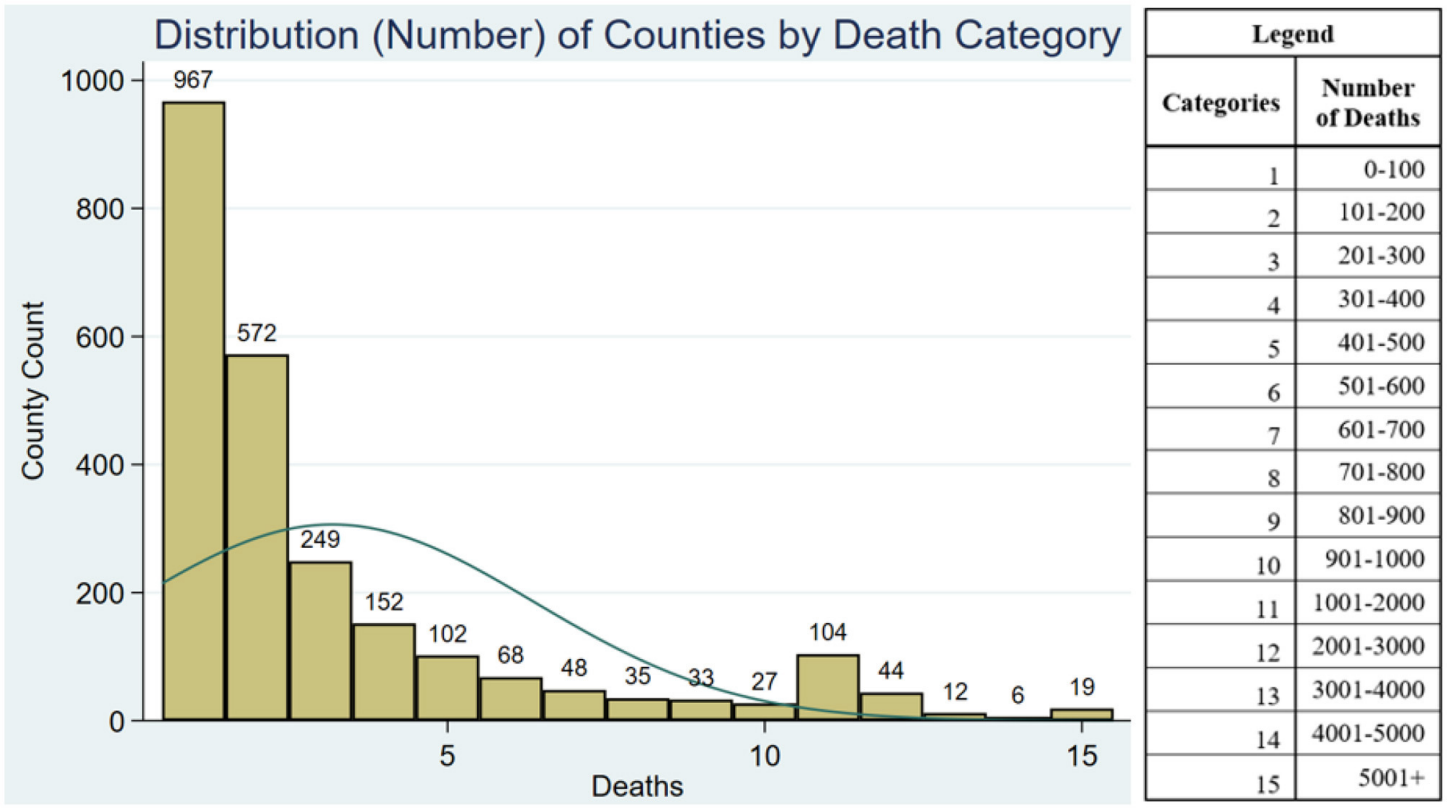
Distribution plots of number of counties by death category. The bars represent the number of counties by death categories. For eg., the first bar (category 1) shows that there were 967 counties with 0 to 100 COIVD-19 deaths.

The descriptive statistics presented in Table 1 shows that there was an average of 387 COVID-19-related deaths across all US counties during the study period (January 22, 2020 to December 14, 2022). Figure 1 shows the distribution plot of the number of deaths by county. This distribution plot shows that our count data, as expected, are skewed to the left, thus justifying the use of a negative binomial regression. Counties in our sample had on average 45.4 FTE RNs per 10,000 population, 1.4 FTE hospitalists per 10,000 population, and 0.3 intensivists per 10,000 population. The average occupancy rate of hospitals across counties was 45.6 and 2.2% of hospitals were teaching hospitals. There were on average 14.4 airborne infection control rooms per 10,000 population. The average county per capita income was $46,770. Urban counties represented 71.1% of our sample. Older adults and racial/minorities represented 19.3 and 24.6% of the counties' population, respectively. There were 284.3 COVID-19 cases per 1,000 population in the county.

**Table 1:** Descriptive statistics of variables included in our study (*n* = 2,348 counties).

**Variables**	**Mean/frequencies**	**Std. Dev**.	**Minimum**	**Maximum**
Deaths	386.96	1,143.46	0	34,006.00
Total county RNs per 10,000 population	45.40	51.14	1.34	1,499.62
Total county hospitalists per 10,000 population	1.39	2.53	0.03	62.53
Total county intensivists per 10,000 population	0.26	0.46	0	5.96
County hospital occupancy	45.55	20.58	0.38	115.47
Proportion of teaching hospitals	2.15	9.87	0	100.00
County airborne infection control rooms per 10,000 population	14.42	95.80	0	3,407.29
Per capita income (in 1,000s)	$46.77	$13.29	$22.44	$229.83
Urban county	71.08%	45.35%	0.00%	100.00%
Percent population 65 years and older	19.29%	4.54%	4.86%	40.80%
Percent racial/ethnic minorities	24.60%	19.81%	2.30%	97.31%
COVID-19 cases per 1,000 population	284.32	65.33	28.38	728.78

The negative binomial regression results assessing the relationship between county-level hospital and macroeconomic resource availability and COVID-19-related deaths are shown in Table 2. Results show that an increase of 10 FTE RN staff per 10,000 population is associated with 3% [IRR = 0.997, CI (0.995–0.999)] decrease of COVID-19-related deaths in the county; an increase of one FTE hospitalist per 10,000 population in the county is associated with a 6.4% [IRR = 0.936, CI (0.897–0.978)] decrease in COVID-19-related deaths, while an increase in one intensivist per 10,000 people in the county is associated with a 39.4% [IRR = 0.606, CI (0.516–0.712)] decrease in COVID-19-related deaths in the county. An increase of 1% occupancy rate in county hospitals is associated with 0.9% [IRR = 1.009, CI (1.003–1.015)] increase in COVID-19-related deaths. An increase of 1% in the proportion of teaching hospitals in the county is associated with a 1.3% [IRR = 1.013, CI (1.008–1.019)] increase in counties' deaths. An increase of one airborne infection control room per 10,000 population in a county is associated with 2.8% increase in the number of COVID-19-related deaths [IRR = 1.028, CI (1.009–1.048)]. A $1,000 increase in per capita income is associated with 1.0% increase in the number of COVID-19-related deaths [IRR = 1.01, CI (1.005–1.015)]. Compared to rural counties, urban counties are associated with a 148.6% increase in COVID-19-related deaths [IRR = 2.486, CI (1.604–3.852)]. A one percent increase in the proportion of racial/ethnic minorities in the county is associated with a 2.7% [IRR = 1.027, CI (1.023–1.031)] increase in COVID-19-related deaths. Finally, we found that 1 case per 1,000 population in the county is associated to a 0.4% increase in the number of COVID-19-related deaths [IRR = 1.003, CI (1.002–1.005)]. Based on these findings, our hypothesis is partially supported.

**Table 2:** Results of negative binomial regression of county-level hospital and macroeconomic resource availability on COVID-19-related deaths (*n* = 2,348 counties).

**Deaths**	**IRR**	**SE**	**(95% CI)**	
Total county RN per 10,000 population	0.997^**^	0.001	0.995	0.999
Total county hospitalist per 10,000 population	0.936^**^	0.021	0.897	0.978
Total county intensivist per 10,000 population	0.606^***^	0.05	0.516	0.712
County hospital occupancy	1.009^**^	0.003	1.003	1.015
County percent teaching hospitals	1.013^***^	0.003	1.008	1.019
County airborne infection control rooms per 10,000 population	1.028^**^	0.01	1.009	1.0483
Thousand per capita income	1.01^***^	0.002	1.005	1.015
Urban county	2.4864^***^	0.555	1.604	3.852
Percent 65+	1.012	0.01	0.992	1.033
Percent minority	1.027^***^	0.002	1.023	1.031
Cases per 1,000	1.003^***^	0.001	1.002	1.005

** p ≤ 0.01.

*** p ≤ 0.0001.

## 6. Discussion

In this study, we explored the associations between county-level hospital and macroeconomic resources and COVID-19-related deaths by analyzing cross-sectional data from 2,438 US counties. The first category pertains to hospital staffing level resources available to hospitals within a county. Staffing variables included total county FTE RNs, hospitalists, and intensivists. As hypothesized, our findings show that a higher county-level staffing level is associated with lower COVID-19-related deaths. These findings are in line with the study by Xie et al. (21). Higher levels of hospital staffing in a county may facilitate the care and treatment of COVID-19 patients, and this in turn has the potential to reduce COVID-19-related deaths. Intensivists play an important role in providing care for the critically ill patients that are admitted in the ICUs. Similarly, in combination with intensivists, hospitalists are valuable resources in treating COVID-19 patients, and even more so in areas where there are no intensivists. As both intensivists and hospitalists create a treatment plan for each patient, nurses are paramount since they are the ones to implement the physician's treatment plan.

The second resource category is county-level care hospital capacity and includes airborne infection isolation rooms and average occupancy rate. When examining our findings on care capacity, one should consider that the availability of care capacity would draw COVID-19 patients; and more patients, particularly the severe ones, would lead to more deaths. In line with this expectation, our findings indicate positive associations between COVID-19-related deaths and total airborne infection isolation rooms and average occupancy rate, in US counties.

Similarly, the positive association between the number of teaching hospitals in a county and COVID-19-related deaths can be explained by the fact that teaching hospitals may attract more severe COVID-19 cases given that these facilities are medical research centers. They have played a critical role during the pandemic by conducting research trials and exploring new therapies. As such, teaching hospitals received a greater amount of critically ill patients, many of which died before appropriate COVID-19 treatments were available. In addition, teaching hospitals often serve multiple counties, leading to inflated number of deaths associated with the hospital's county.

As hypothesized, we find that staffing resources are associated with lower COVID-19 deaths. However, facility resources (like bed capacity, teaching hospitals, and county airborne infection control rooms) are associated with higher COVID-19 deaths. The positive relationship between facility resources and deaths can be explained by the increased demand particularly from sicker patients that these counties may have as a result of their higher capacity. Policymakers may want to focus on staffing resources as a mechanism to address future pandemics. However, increased facility resources may be needed in counties with lower capacity.

The results of our study are consistent with previous studies that demonstrated that urban counties are associated with higher COVID-19-related deaths compared to rural counties (22), possibly because urban counties were the first and hardest hit counties. Our findings show per capita income is associated with higher COVID-19-related deaths. Counties with higher per capita income have better healthcare facilities and resources to better care for COVID-19 patients, which may attract patients from neighboring counties with fewer resources seeking care. More COVID-19 patients may potentially lead to higher deaths. Additionally, people with more resources are more likely to travel and potentially contract the virus during their trip, increasing Covid-19 cases and related deaths in the county (23). Similarly, our study is supported by other studies that have found increased COVID-19-related deaths among racial/ethnic minorities (24). It has been reported in the literature that this association could be due to the high prevalence of certain maladies such as cardiovascular diseases, diabetes, pulmonary disease, hypertension, among minorities (25–27).

### 6.1. Limitations

Our study has some limitations. First, we used secondary data, which may involve some issues in data reliability. However, our data sources are widely used in health services research. Second, data limitation precluded us from including resources such as ventilators, ambulances, paramedics, pulmonologists, medications, personal protective equipment (PPE), and other medical supplies, as well as the existence of a disaster preparedness plan. Concerning PPE, to our knowledge, there is no nationwide data repository, let alone county level data on availability of PPE. This situation has been stated by Dr. Raja on Give Us PPE who reported that “There is still no centralized federal database for personal protective equipment. The disconnect between what's purported to be in the stockpile and the needs on the front-line is astounding” (28). More studies are needed to determine the relationship between healthcare resources and COVID-19-related deaths. Third, our resource data were from 2019, while the COVID-19-related death data were from 2020 to 2022. Although resource data are not likely to change much from year to year, future studies should explore these relationships with more recent resource data. Finally, our study is cross-sectional; therefore, we cannot imply causal relationships. Longitudinal studies are needed in the future to examine the relationship between resource availability and pandemic outcomes.

## 7. Conclusion

This study makes a significant contribution to the literature by exploring the association between county-level hospital and macroeconomic resources and COVID-19-related deaths in US counties. To our knowledge, this is among the first comprehensive studies examining the role of hospital and macroeconomic resources on COVID-19 deaths. Our findings show that the availability of hospital staffing, particularly nurses, hospitalists, and intensivists, is associated with lower COVID-19-related deaths. However, county-level hospital capacity in the form of airborne infection isolation rooms, increased occupancy rate, and a higher proportion of teaching hospitals are associated with higher COVID-19-related deaths. The positive relationship between capacity and COVID-19-related deaths may be simply a reflection of the higher demand and increased inpatient admissions of more acutely ill COVID-19 patients to hospitals in those counties. Finally, consistent with prior studies, urban counties, and a higher proportion of racial/ethnic minorities were associated with higher COVID-19-related deaths.

### 7.1. Implication for policy and practice

Stakeholders of the COVID-19 pandemic, including hospital administrators, clinicians, public-health experts, and policymakers, may use our study findings to develop strategies or interventions to mitigate COVID-19-related deaths and better prepare for future pandemics. This is particularly important as it pertain to the availability of human resources such as nurses, intensivists, pulmonologists, hospitalists, and paramedics, which are critically important to save lives. In addition to human resources, capital resources such as ventilators, airborne infection control rooms, and ambulances are important resources in a pandemic. Other resources not included in our study such personal protective equipment (PPE), medications, and vaccinations have also been reported to be important in addressing a pandemic (29, 30). The development of a disaster preparedness plan focused particularly on novel viruses, will be very important. The lack of PPE, particularly necessary to help protect those who were on the front line trying to save the lives of the vulnerable gave an impression of our health care system either not being prepared, or not able to implement the existing plan to face this novel ailment and others that may appear in the future. As suggested by Dr. Raja (28), given the mishap experience particularly at the onset of the pandemic, a recommendation would be to have available data on PPE at the federal level to be able to track availability nationally and by county.

## Data availability statement

Publicly available datasets were analyzed in this study. These data can be found here: American Hospital Association Annual Survey of Hospitals (https://www.aha.org/topics/hospital-data); Area Health Resource Files (https://data.hrsa.gov/data/download?data=AHRF#AHRF); USA Facts (https://usafacts.org/visualizations/coronavirus-covid-19-spread-map).

## Author contributions

Conceptualization, methodology, writing-original draft preparation, and writing-review and editing: JE, FZ, ZR, LM, and RW-M. Validation: RW-M. Formal analysis: JE. Resources: JE, FZ, and RW-M. Data curation: JE. All authors have read and agreed to the published version of the manuscript.
